# A Bilayer Feature Fusion Framework for Pan-Cancer Survival Prediction Based on Multihead Attention and Adaptive Differential Privacy: Model Development and Validation Study

**DOI:** 10.2196/83743

**Published:** 2026-03-30

**Authors:** Yun Chen, Zhifang Deng, Lili Wang, Huanhuan Wang, Xiang Wu

**Affiliations:** 1 School of Medical Information and Engineering Xuzhou Medical University Xuzhou China; 2 Yunlong Lake Laboratory of Deep Underground Science and Engineering Xuzhou China; 3 The Affiliated Hospital of Xuzhou Medical University Xuzhou China

**Keywords:** adaptive differential privacy, multihead attention, pan-cancer survival prediction

## Abstract

**Background:**

In the field of precision medicine, pan-cancer survival prediction is crucial for individualized oncology diagnosis and treatment. Although multimodal data fusion techniques have significantly improved prediction accuracy, existing studies generally overlook the sensitivity of medical data and the need for privacy protection.

**Objective:**

To address the aforementioned problem, this study aims to propose a bilayer feature fusion framework based on the multihead attention mechanism and adaptive differential privacy, which balances precise feature extraction and sensitive data protection.

**Methods:**

Specifically, the multihead attention mechanism was integrated into bilayer feature extraction and fusion. Layer-wise relevance analysis was used to calculate the correlation between features and outcomes, and Laplacian noise was adaptively added based on the calculation results to achieve collaborative optimization of precise feature extraction and sensitive data protection. Additionally, the concordance index (C-index) and 5-fold cross-validation were used to compare the proposed method with state-of-the-art approaches.

**Results:**

The proposed model achieved superior performance in both pan-cancer and single-cancer survival prediction, validated via the C-index and 5-fold cross-validation. In pan-cancer scenarios, the trimodal combination of clinical data, messenger RNA data, and microRNA expression data achieved the highest C-index of 0.799, outperforming multiple existing multimodal survival prediction approaches. After adaptive Laplacian noise injection for privacy protection, the model’s accuracy decreased by only 0.01-0.03 while satisfying ϵ-differential privacy. For single-cancer prediction, the proposed method achieved higher C-index values in 18 out of 20 cancer types compared with representative deep learning–based survival models, with 7 types showing significant improvements (C-index difference >0.1) and more stable performance distributions. Furthermore, pan-cancer–trained models generally outperformed single-cancer–trained counterparts in most cancer types, highlighting the value of shared predictive features across cancers.

**Conclusions:**

This study provides a solution that balances prediction accuracy and privacy security for pan-cancer survival prediction, laying the foundation for the efficient use of medical data under privacy protection. Future work may further integrate pathological images and proteomics data to expand the model’s applications in cancer subtype classification and biomarker discovery.

## Introduction

In the era of precision medicine, pan-cancer survival prediction refers to the task of modeling and predicting patient survival outcomes across multiple cancer types simultaneously by leveraging shared and cancer-specific prognostic patterns. As a core component of individualized oncology diagnosis and treatment, pan-cancer survival prediction has been widely validated for its clinical value. Accurately estimating survival risk for patients with different cancer types not only enables clinicians to optimize treatment strategies and personalize follow-up schedules, but also facilitates risk stratification and more efficient allocation of medical resources [1].

Early survival prediction efforts were predominantly anchored in unimodal data paradigms, relying on either genomic features or clinical variables in isolation. Statistical models laid the foundational groundwork for this field: the random survival forest (RSF) [2], for instance, addressed key challenges in survival analysis (eg, right-censored data and missing values) through log-rank–based tree splitting and adaptive imputation, establishing a robust nonparametric framework. Meanwhile, the classic Cox proportional hazards model (CPH) [3] became a clinical staple for its interpretability via linear combinations of clinical indicators, though its inability to capture complex nonlinear biological mechanisms limited its predictive power in heterogeneous cancer cohorts. The advent of deep learning catalyzed a paradigm shift in unimodal modeling: approaches like DeepSurv [4] integrated neural networks with Cox regression to automatically learn nonlinear covariate–treatment relationships, outperforming traditional models on genomic datasets. Subsequent studies further validated deep learning’s superiority in capturing complex variable interactions; for example, in clinical data for oral squamous cell carcinoma [5] and histopathological images for colorectal cancer [6]. Weakly supervised learning also emerged as a promising direction for leveraging unannotated whole-slide images across multiple cancer types [7], while transfer learning facilitated cross-dataset generalization in disease-specific prognostic tasks [8]. Despite these advances, unimodal models inherently suffer from incomplete information capture: genomic data alone overlooks the spatial heterogeneity of tumor microenvironments (eg, angiogenesis patterns in pathology slides), while clinical variables fail to reveal molecular-level prognostic markers, creating an inherent ceiling for predictive performance.

In response to these limitations, multimodal data fusion has emerged as a transformative research focus in pan-cancer survival prediction, driven by the recognition that complementary data modalities (omics, imaging, and clinical) can synergistically enhance prognostic accuracy. Early multimodal frameworks integrated clinical, transcriptomic (messenger RNA [mRNA]/microRNA [miRNA]), and pathological data to achieve cross-cancer prediction [9], while recent innovations have incorporated advanced techniques such as unsupervised learning and attention mechanisms to dynamically weight modality contributions [10]. A key trend in this space is the development of specialized fusion architectures: the Multimodal Affinity Fusion Network [11] and MultiCoFusion [12]; for example, use attention mechanisms and graph convolutional networks to model intermodal dependencies and gene-gene interactions, respectively. Furthermore, bilinear fusion approaches, such as the attention-based multimodal bilinear fusion [13] and hierarchical factorized models [14], have addressed the curse of dimensionality to enable deeper cross-modal interactions. Notably, methods like Dynamic-DeepHit [15] have pioneered the use of recurrent neural network–based attention to model longitudinal dependencies for dynamic risk prediction, while cross-modal translation and alignment (CMTA) [16] explicitly learn and aligns intra- and cross-modal representations between pathology and genomics via encoder-decoder structures. End-to-end multimodal models such as MultiSurv [17], which integrate up to 6 modalities (whole-slide images, clinical data, and multiomics), have demonstrated state-of-the-art performance (concordance index [C-index]=0.779) across 33 cancer types, validating the value of comprehensive data integration. Other frameworks have incorporated multitask learning [18] or alternating training [19] to jointly optimize survival prediction and auxiliary tasks (eg, cancer grading), further improving model robustness. Recent work by Flack et al [20] introduced a robust multimodal survival prediction framework that effectively handles data heterogeneity and missing modalities through a regularization-based fusion strategy, showing competitive performance across multiple cancer types. Despite these advancements, critical challenges persist: inefficient cross-modal information alignment, inadequate intramodal feature refinement, and unresolved issues of intermodal heterogeneity and noise propagation remain major barriers to achieving consistent performance across diverse cancer types at the pan-cancer scale. More critically, the majority of these state-of-the-art multimodal survival prediction models, including MultiSurv [17], HFBSurv [14], attention-based multimodal bilinear fusion [13], and MBFusion [18], operate under the implicit assumption of centralized, nonsensitive data. They fundamentally lack integrated mechanisms to protect the highly sensitive genetic and clinical information inherent in medical datasets, creating a significant gap between methodological advancement and real-world clinical applicability where privacy is paramount.

Although multimodal data fusion has significantly improved pan-cancer survival prediction accuracy, existing research generally ignores the sensitivity of medical data and the need for privacy protection. Medical data contain sensitive information such as patients’ genomic data, pathological images, and clinical records, whose leakage may lead to ethical risks such as identity recognition and insurance discrimination. For instance, empirical studies have demonstrated that even aggregated genomic data remain vulnerable to linkage attacks using auxiliary public databases, potentially inferring an individual’s identity with high confidence [21]. Additionally, during the training and sharing of deep learning models, gradients or intermediate features may inadvertently leak sensitive patterns of the original training data [22]. While Chen et al [23] proposed an optimized logistic regression model (batch gradient descent logistic regression and balanced differential privacy logistic regression based on hybrid feature selection (Pearson correlation test + random forest out-of-bag algorithm) and differential privacy (DP) protection to address the problems of insufficient prediction accuracy and privacy leakage in existing machine learning models for breast cancer prediction, and Chai et al [24] proposed the decentralized federated learning framework AdFed, combining regularization methods to train models without sharing raw data while achieving feature selection and privacy protection. To protect DNA data, Wu et al [25] proposed the DP-Motif Finding algorithm based on ϵ-DP, which uses closed frequent patterns to reduce redundant motifs, allocates privacy budget by constructing a perturbed extension tree, and uses best linear unbiased estimation postprocessing to optimize the noisy support. Wu et al [26] also proposed an adaptive federated learning scheme integrating DP, which adjusts the gradient descent process through an adaptive learning rate algorithm to avoid model overfitting and fluctuations, while introducing a DP mechanism to resist various background knowledge attacks, providing quantifiable privacy protection for the federated learning process. Wang et al [27] proposed a blockchain-based access control framework for genome-wide association studies in federated learning to protect the security of genetic data—this framework implements automated quality control to ensure training data quality, designs a blockchain-based authentication mechanism to filter malicious attackers, and adopts a periodic aggregation method combined with DP to accelerate cloud model training and resist multiple attacks [28]. Wang et al [29] proposed a method based on the ant colony optimization algorithm to detect gene interactions for genome-wide association studies—an intelligent privacy-preserving scheme. Current studies still suffer from problems such as single datasets and heavy reliance on data anonymization or centralized storage, failing to embed dynamic privacy protection mechanisms.

To solve the above problems, this study proposes a bilayer feature fusion framework based on the multihead attention (MHA) mechanism and adaptive DP, achieving collaborative optimization of precise feature extraction and sensitive data protection through technological innovation. Specifically, for feature fusion, traditional methods typically rely on fixed weights or simple concatenation to handle heterogeneous data. Simple concatenation, however, merely “physically stacks” feature vectors from different modalities, posing notable limitations. Multisource data (clinical, mRNA, miRNA, and gene copy number variation [CNV]) vary drastically in feature dimensions, semantics, and distributions; forcing them into a single-vector space postconcatenation overlooks inherent intermodal correlations; for example, links between specific mRNA expression and clinical survival time, or regulatory relationships between miRNA and CNV. Additionally, concatenation assumes “all features are equally important,” failing to differentiate their contributions to survival prediction. This often buries key signals (eg, driver gene mutation sites) under redundancy, and may trigger the curse of dimensionality via rapid feature expansion, increasing computational complexity and overfitting risk. Given the aforementioned limitations of simple concatenation, our first contribution is the design of a structured bilayer feature extraction module. The first layer performs modality-specific feature extraction on clinical, mRNA, miRNA, and CNV data through fully connected (FC) networks and embedding layers. The second layer innovatively uses an MHA mechanism not merely as a fusion tool, but as a structured cross-modal interaction model that explicitly quantifies feature-level contributions. Unlike single-head attention, which models only single correlations, MHA captures differentiated correlations between features from different subspaces through parallelized independent attention heads and focuses on key survival-related features through dynamic weight allocation. Our second and primary contribution lies in the novel integration of adaptive DP directly into the multimodal feature extraction pipeline. At the privacy protection level, this study proposes adaptive DP based on layer-wise relevance analysis, balancing prediction accuracy and privacy security through a 2-step strategy. First, the layer-wise relevance propagation algorithm is used to quantify the association strength between neurons at each layer and survival prediction outcomes, identifying highly sensitive features (eg, driver gene mutation sites) in mRNA, miRNA, and CNV data; then privacy budgets are dynamically allocated based on correlation scores to inject Laplacian noise into the gradient update process—features with higher correlations receive less noise, preserving key prediction information while suppressing privacy leakage risks.

In summary, the novelty of our work is not merely the combination of MHA and DP, but the creation of a synergistic framework where (1) the bilayer MHA structure provides the granular feature importance signals necessary for adaptive noise allocation and (2) the adaptive DP mechanism, guided by layer-wise relevance analysis, protects privacy in a targeted manner that minimizes damage to the model’s core predictive capability. This integrated solution addresses the specific limitations of prior multimodal models (which neglect privacy) and prior DP applications (which impair use in complex models), paving the way for clinically viable, privacy-preserving pan-cancer prediction tools.

## Methods

### Overview

**Figure 1:**
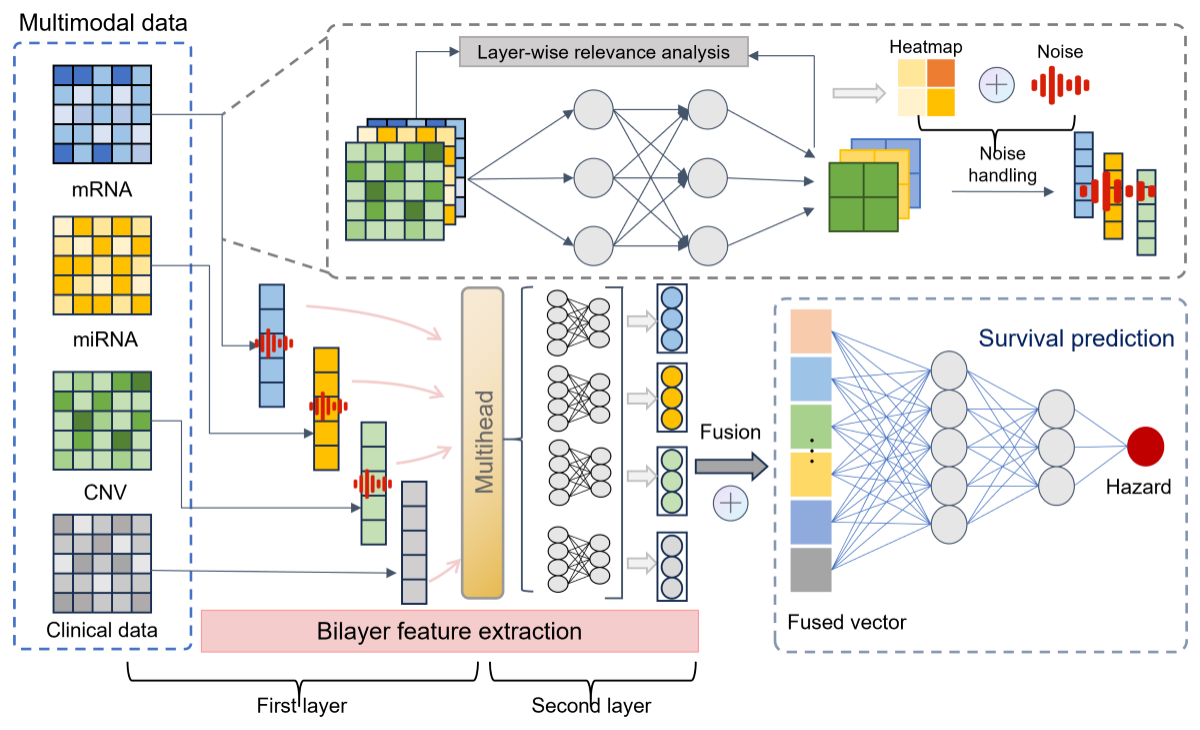
The architecture of the proposed model. CNV: gene copy number variation; miRNA: microRNA; mRNA: messenger RNA.

As shown in Figure 1, the proposed model architecture consists of 2 parts: a bilayer feature extraction module based on MHA and an adaptive DP protection module based on layer-wise relevance analysis. The bilayer feature extraction part, based on MHA, first performs first-layer feature extraction through FC layers and embedding layers, and then uses the MHA mechanism in the second layer for further feature extraction and multimodal data fusion. By integrating the MHA mechanism, the bilayer feature extraction module fully uses the advantage of parallel heads to capture different correlations, comprehensively and deeply mining key information from multimodal data. Additionally, a privacy protection component is added during the first-layer feature extraction, integrating layer-wise relevance analysis into the FC layers to deeply analyze the correlation between features and survival prediction outcomes through backpropagation. Noise is adaptively added based on the obtained correlation results. This study introduces a bilayer feature extraction module into the multimodal survival prediction model to better mine data features and provides privacy protection, effectively preserving the privacy information in the training data. The data preprocessing and detailed method descriptions are presented in the following sections.

### Data Preprocessing

In this study, 4 types of data were used: clinical data, gene expression (mRNA) data, miRNA data, and CNV data.

This section performs data screening for different data features. For clinical features, patient data with missing values and/or missing follow-up times were excluded. For gene expression data, a variance threshold method was used to select features with variances greater than a given threshold calculated from all patients [18,30]. In this study, the same thresholds as Fan et al [10] were used for mRNA and CNV modalities: 7 and 0.2, respectively. This processing retained 1579 mRNA features and 2711 CNV features. Subsequently, all continuous variables were normalized to the interval (0, 1) using minimum-maximum normalization [31].

Considering the variations in diagnosis and treatment methods across different cancer types, samples in the dataset may lack some required modalities. This study adopts zero-vector imputation for missing modalities, with its rationale mainly reflected in 2 aspects: first, zero vectors can explicitly indicate “no measured data for the modality,” avoiding false biological signals introduced by statistical imputation methods. Second, zero-vector imputation ensures the consistency of feature dimensions across all samples, simplifies the model’s input structure, and avoids additional computational overhead from complex missing-value processing modules, providing stability guarantees for gradient updates in the integration of adaptive DP mechanisms.

### Bilayer Feature Extraction Based on MHA

**Figure 2:**
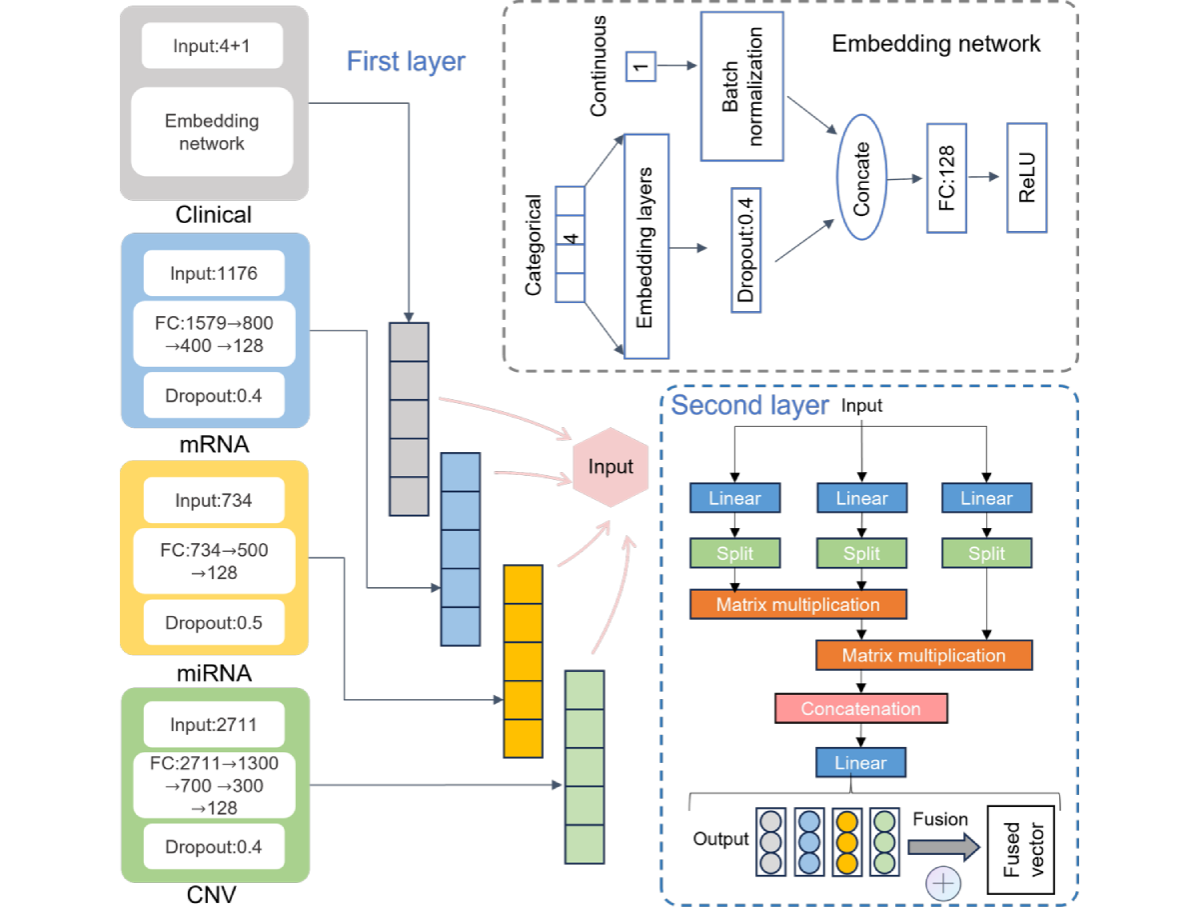
The architecture of bilayer feature extraction based on multihead attention. CNV: gene copy number variation; FC: fully connected; miRNA: microRNA; mRNA: messenger RNA; ReLU: rectified linear unit.

To improve the accuracy and efficiency of feature extraction, this study designs a bilayer feature extraction method based on MHA, which can more comprehensively and deeply mine key information from multimodal data, as shown in Figure 2.

In recent years, neural networks with FC layers have been widely used for representation extraction [15]. In this study, different neural network settings with FC layers were used as the first-layer feature extraction to obtain representations from the raw numerical data of different modalities. Batch normalization was adopted to normalize the layer inputs and accelerate neural network training [30]. For the clinical data, we used categorical embedding layers for the 4 categorical variables, namely, cancer type, gender, race, and histological type, with dropout to encode the categorical variables into a numerical vector. The normalized continuous variables (ie, age) were then concatenated with these numerical vectors and fed into an FC layer with a fixed representation length. For miRNA, mRNA, and CNV modalities, 2-4 FC layers with rectified linear unit activation and batch normalization were used to extract fixed-length representations. The representation vectors 
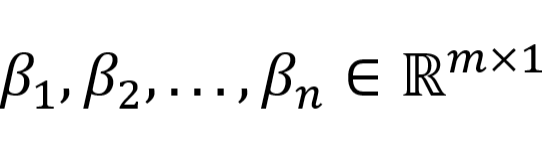
 were obtained from modality-specific configurations to form the representation matrix 
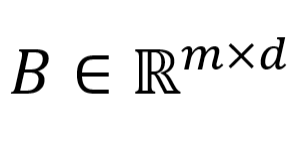
 where *m* is the sample size and *d* is the feature dimension.

To effectively capture the complex interaction relationships between multimodal data and enhance feature representation capabilities, the MHA mechanism was introduced in the second-layer feature extraction stage.

The proposed MHA mechanism consists of 3 components similar to the self-attention in the transformer model: query (*Q*), key (*K*), and value (*V*). The matrix *B* is projected to generate keys *K* and values *V*. Specifically, *V* is processed using an identity function because the features extracted in the first layer already have sufficient representational power. For *K*, it is generated by:



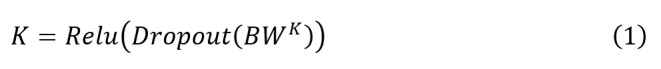



Where 
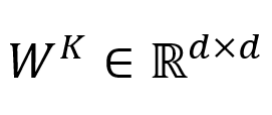
 (*d* represents the feature dimension) is a trainable matrix randomly initialized from a standard Gaussian distribution and updated during training; *B* is the representation matrix obtained from the first-layer feature extraction. The dropout layer and rectified linear unit activation function are used to prevent overfitting and introduce nonlinearity. The query 
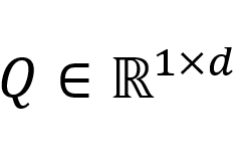
 is a learnable vector initialized randomly, with elements sampled from a uniform distribution 
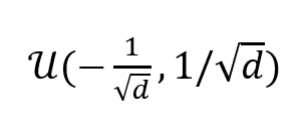
.

The attention aggregation feature is calculated as:



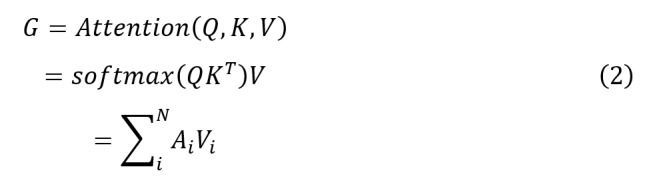



Where *A* is the attention weight. After softmax normalization along the sample dimension, the sum of the attention weights *A* for *N* samples is 1, enabling dynamic focusing on key survival-related features.

To capture multidimensional survival associations, the attention layer is extended to *H* heads. Specifically, *Q*, *K*, and *V* are split into blocks of size *d*/*H* along the embedding dimension. The attention for each head is calculated as:



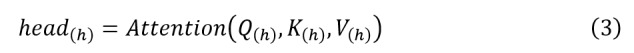



These heads are then concatenated into a multihead output:



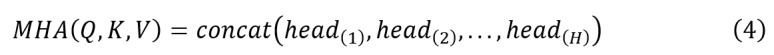



This process allows different heads to capture interactions between different types of data separately.

Finally, the fused features are obtained by:



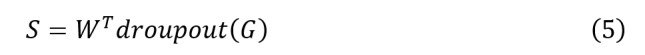



Where 
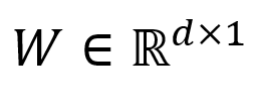
 is the weight of the last layer. In evaluation mode, the dropout layer is disabled. Thus, the fused feature *Y* can be written as:



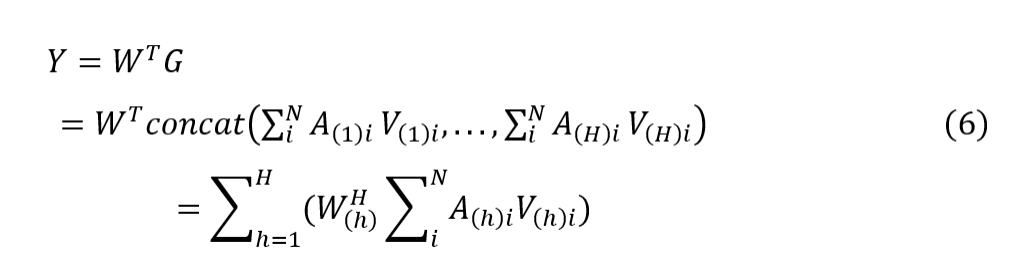



The attention mechanism focuses on key information through dynamic weight allocation, efficiently handling long-range dependencies in sequence data and suppressing noise interference. However, its single-head structure has limitations in modeling multidimensional correlations. Therefore, based on the features extracted from the 4 modalities in the first layer, this study introduces the MHA mechanism. By parallelizing multiple independent attention heads, it captures differentiated correlations between features from different subspaces, providing more comprehensive feature representations for pan-cancer survival prediction. The above method achieves deep fusion of multimodal survival features, offering high-precision feature representations for pan-cancer survival prediction.

### Adaptive DP Based on Layer-Wise Relevance Analysis

In the field of cancer survival prediction, privacy protection of medical data is of utmost importance. Genetic data, such as mRNA, miRNA, and CNV data, contain highly sensitive personal information, posing a risk of privacy leakage. Therefore, during the feature extraction process, this study designs a method based on layer-wise relevance analysis to construct a noise addition mechanism by adding Laplacian noise for privacy protection, ensuring data security.

**Figure 3:**
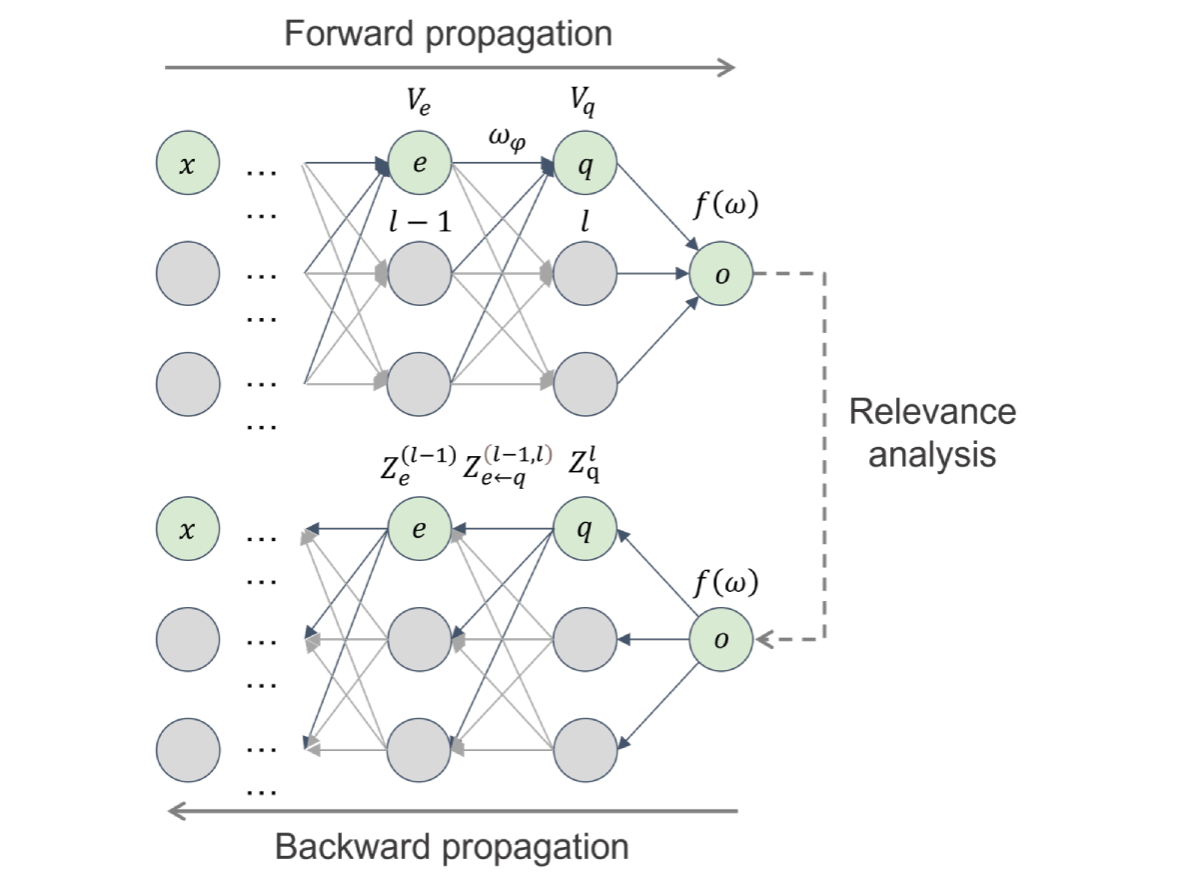
The process of the layer-wise relevance analysis method of the proposed model based on the backpropagation algorithm.

First, during the extraction of mRNA, miRNA, and CNV features in the first layer, this paper adds appropriate Laplace noise to the training gradient of neurons based on analyzing the correlation between each neuron layer and the output layer. The core theoretical operation process is shown in Figure 3. Relevance analysis begins after forward and backward propagation, calculating the correlation results of each feature in a layer of neurons. Importantly, the correlation analysis results are adjusted with the forward and backward propagation processes until the propagation ends.

The correlation between each input feature *x_ij_* and the output *Hx_i_*(*0*) is calculated by decomposing the neurons of the previous layer. Given *Z_e_*^(^*^l^*^)^(*x_i_*) as the correlation resul
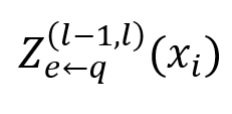
 t between *x_i_* and neuron *e* in layer *l*, define as the process of neuron *e* sending information to *q*. The neuron correlation is:



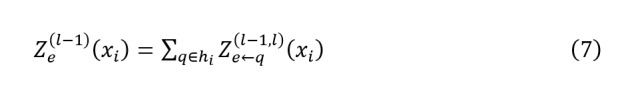



The decomposition rule for layer-wise relevance analysis is:



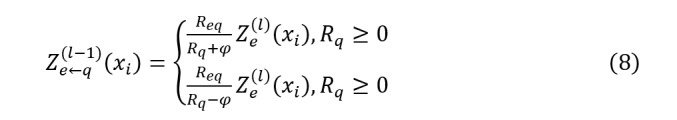



Where is a predefined stabilizer to address the unboundedness of *Z_e_*^(^*^l^*^)^(*x_i_*). *R_q_* is the affine transformation of neuron *e*, defined as:



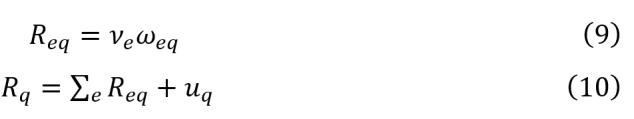



Where *v_e_* is the value of neuron *e*,*_eq_* is the weight between neuron *e* and *q*,*u_q_* is the bias term.

In the last hidden layer, for the output variable *o*, the correlation is calculated as:



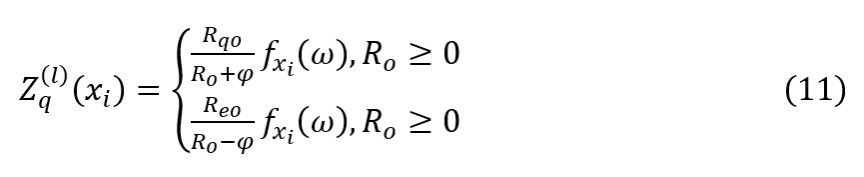



This study directly calculates the correlation score *Z_j_*(*x_ij_*) between each feature *x_ij_* inlayer *j* and the survival prediction result for mRNA, miRNA, and CNV features.

Next, a noise addition rule is defined. Through the above layer-wise relevance analysis, the value range of these correlation scores is divided into *n* nonoverlapping threshold intervals 
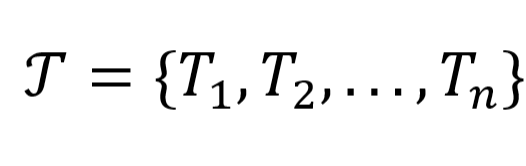
, and *T*_1_ corresponds to the features with the highest relevance scores.

A privacy-budget mapping function 
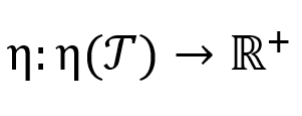
 is then defined, which assigns a specific privacy parameter *ϵ_k_* to each interval *T_k_*. The function is designed to be strictly monotonic, satisfying the following condition: for any *g*<*h*,*η*(*T_g_*)<*η*(*T_h_*), thereby ensuring that features with stronger predictive correlations are allocated a higher privacy budget (less noise), while features with weaker correlations receive a lower privacy budget (more noise).

Formally, for an interval index *k*∈{1,2,...,*n*},the privacy parameter *ϵ_k_* is given by:



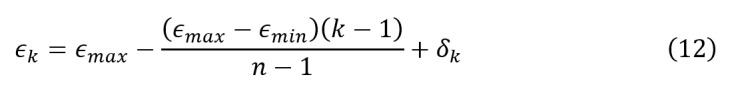



Where *ϵ_max_*=0.5 and *ϵ_min_*=0.1 define the permissible range of the privacy budget; *δ_k_*~*U*(–∆,∆) is a bounded random perturbation term that preserves the strict monotonicity *ϵ*_1_>*ϵ*_2_>...>*ϵ*_n_.

Privacy budgets are adaptively allocated according to the function :



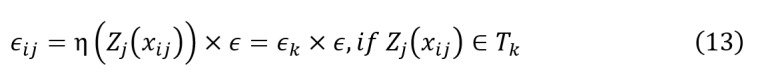



Where *ϵ* denotes the global privacy budget.

Laplacian noise is added to the training gradients of neurons, where *ϵ* is the total privacy budget for protecting mRNA, miRNA, and CNV features.

At the start of training, the gradient update objective function of the general optimization method is defined. In each training step, using a set of random training samples *L* from the genetic data feature set *X* (where *X* = {*x*_11_, *x*_12_,..., *x_ij_*}), starting from the initial point _0_, the parameters are updated at step *t* as:



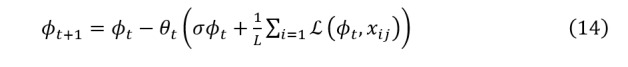



where *ϴ* is the learning rate at step *t*, is the regularization parameter, and 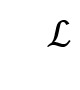 is the loss function.

Subsequently, the training gradients are perturbed to ensure the security of genetic data during training and sharing:



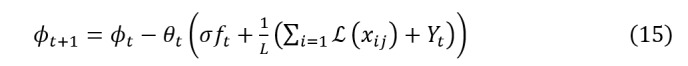



Where *Y_t_* is the Laplacian noise.

After detailing the process of adding Laplacian noise to features based on layer-wise relevance analysis, this section proceeds to prove the implementation of ϵ-DP. The following proof shows how the proposed method satisfies ϵ-DP requirements:

*Proof*: assume *L* and *L*’ are 2 adjacent batches, and *_t_*_+ 1(_*_L_*_)_ and *_t_*_+ 1(_*_L’_*_)_ are the parameters for *L* and *L*’, respectively. The formula is expressed as follows:



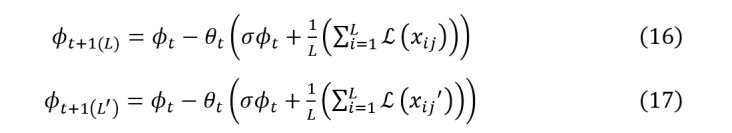



Then, the inequality for the difference between the 2 output results is:



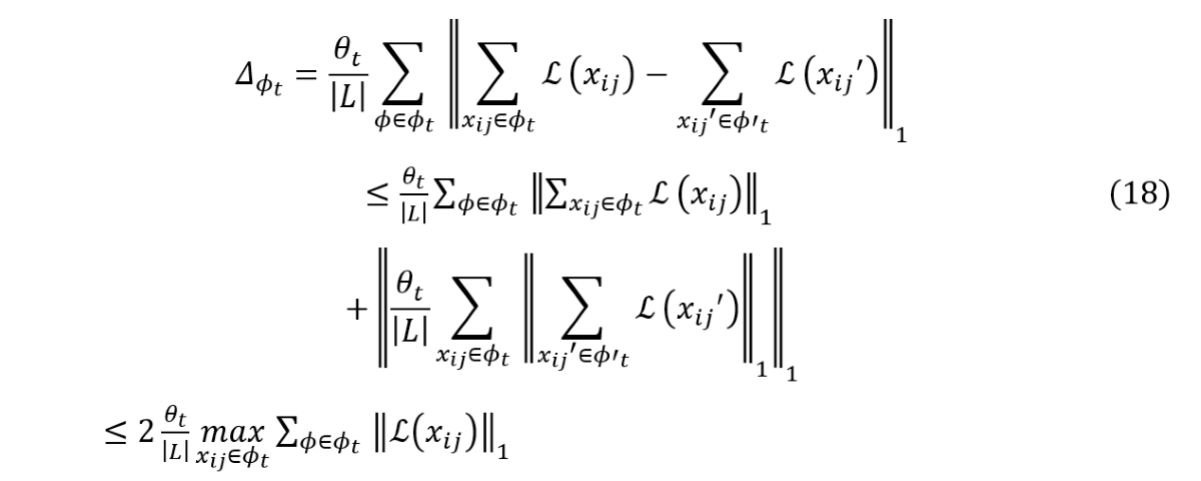



From the above formula and DP, *_t_* is the sensitivity of the neural network **Inline graphic 27**. To protect the privacy information of the neural network, the gradient is perturbed based on relevance analysis, and the noise can be written as:



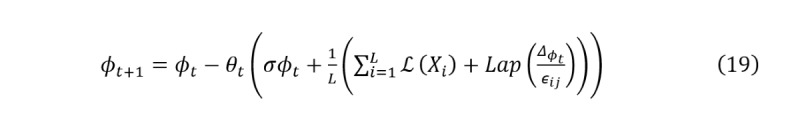



We have:



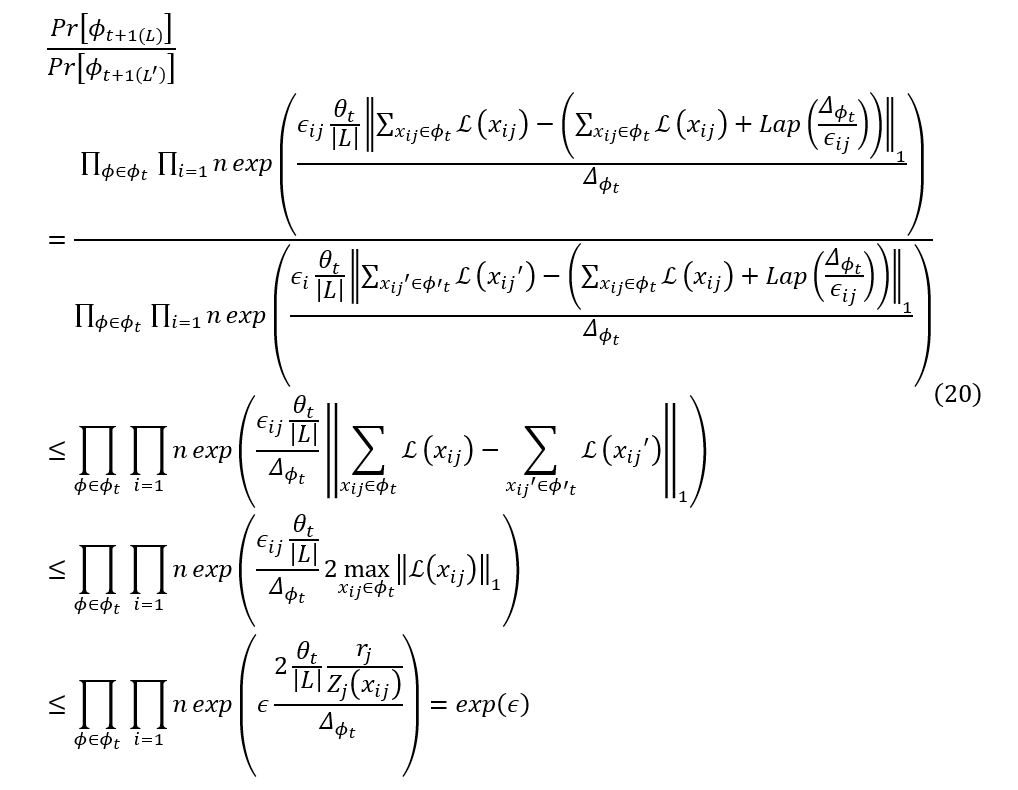



This proves that the method satisfies ϵ-DP, ie,


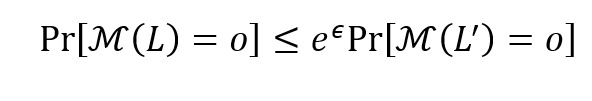
, where *M* is the model after adding Laplacian noise to features. It ensures that the model’s noise addition mechanism is consistent with the definition of DP while protecting the privacy of mRNA, miRNA, and CNV features.

This method not only effectively reduces the risk of data leakage but also maximizes data usability, providing a more secure and reliable solution for cancer survival prediction and promoting the efficient use and sharing of medical data under privacy protection.

### Ethical Considerations

All data used in this study were obtained from The Cancer Genome Atlas (TCGA), a publicly available database. The TCGA project obtained informed consent from all participants and received ethical approval from the appropriate institutional review boards. As this study involved only the use of deidentified, publicly accessible data, no additional ethical approval was required.

## Results

### Data

The clinical, mRNA, and miRNA data were downloaded from the Pan-Cancer Atlas of TCGA project (publicly accessible at the Genomic Data Commons), where more than 11,000 patient samples across 33 tumor types [32,33] were collected. The clinical dataset provides annotations for 11,160 patients, using 5 variables consistent with Silva and Rohr [17]: cancer type, gender, race, histological type, and age. The miRNA data set contains 743 miRNA feature records, and the mRNA dataset contains RNA sequencing counts for 20,531 mRNAs. The CNV data were downloaded from the University of California Santa Cruz Xena [34].

Table 1 describes the data information after preprocessing in detail. In this study, the pan-cancer datasets were partitioned into training (6656/11094, 60%), validation (2219/11094, 20%), and testing (2219/11094, 20%) subsets, with model performance evaluated using 5-fold cross-validation.

**Table 1:** Summary information of the different modalities after preprocessing.

Modality	Patients, n	Features, n		All zero vector, n/N (%)
		Continuous	Categorical	
Clinical	11,094	1	4	—^a^
miRNA^b^	11,094	743	—	72/743 (9.7)
mRNA^c^	11,094	1579	—	49/1579 (3.1)
CNV^d^	11,094	2711	—	225/2711 (8.3)

^a^Not available.

^b^miRNA: microRNA.

^c^mRNA: messenger RNA.

^d^CNV: gene copy number variation.

### Experimental Settings

We conduct all experiments on a simulation environment equipped with a 64-bit Intel(R) Xeon(R) Silver 4210R CPU @ 2.40 GHz processor, 32 GB RAM, and an NVIDIA GeForce RTX 3080 GPU for accelerated computation. The experiments are implemented on the Windows 11 operating system, with Python (version 3.10; Python Software Foundation) as the primary programming language and PyTorch (version 2.6.0; Meta AI) as the deep learning framework.

For model training, the Adam optimizer is adopted with a fixed learning rate of 0.001. Key hyperparameters are configured as follows: sequence length is set to 128, batch size is 256, and total training epochs are 100.

### Evaluation Metrics

In this study, we assessed the performance of our model by using the C-index, a widely adopted metric for evaluating survival predictions in censored survival data [2,35]. The C-index measures the proportion of concordant pairs among all possible evaluation pairs, defined as:



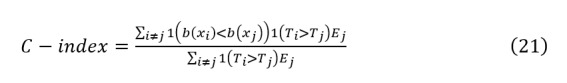



where 1 is the indicator function of whether the expression in parentheses is true or false. The C-index ranges from 0 to 1. The closer the C-index is to 1, the closer the prediction order is to the real one; the closer the C-index is to 0.5, the closer the model's prediction is to a random prediction. Notably, the C-index focuses on the ordinal relationship of predictions rather than the accuracy of individual sample forecasts, making it particularly suitable for evaluating proportional hazards models.

Accuracy was also used for performance evaluation. The metric is evaluated as follows:



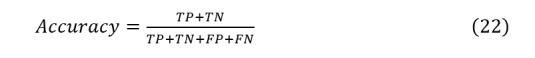



### Layer-Wise Relevance Analysis

To evaluate the model’s performance in privacy preservation, this study determines the privacy budget through hierarchical correlation analysis and subsequently adds Laplacian noise based on the results. Specifically, this study analyzed the strength of correlations between features and different FC layers using heatmaps for the 3 modalities: mRNA, miRNA, and CNV. The computed correlation scores are sorted in descending order, and the feature scores at the 25th, 50th, 75th, and 100th percentiles are visualized. The heatmaps show that higher correlation scores indicate stronger associations between the corresponding features and survival prediction outcomes.

**Figure 4:**
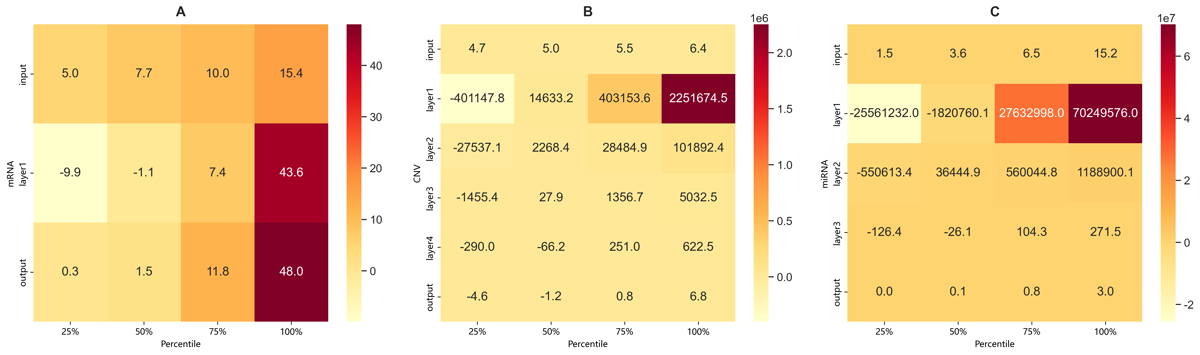
Heatmaps of correlation scores between layers and features in (A) messenger RNA (mRNA), (B) gene copy number variation (CNV), and (C) microRNA (miRNA) modalities.

A more detailed analysis of each modality’s heatmap reveals that, as shown in Figure 4A, the output layer and mRNA layer 1 exhibit significantly higher correlation scores at the 100th percentile (48.0 and 43.6, respectively), with notable increases compared to the 25th, 50th, and 75th percentiles. This suggests that these 2 layers are particularly effective in capturing high-correlation features, which are strongly associated with survival outcomes. These findings indicate that privacy-preserving noise injection should focus on these layers to avoid the leakage of critical predictive information.

For the CNV and miRNA modalities, as illustrated in Figures 4B and 4C, layer 1 demonstrates markedly higher correlation scores at the 100th percentile compared to subsequent layers and the output layer. This suggests that the model primarily extracts key features from layer 1, with diminishing correlation strength in deeper layers. Therefore, noise injection strategies should prioritize high-correlation features in layer 1, enhancing privacy protection while maintaining the model’s feature extraction performance.

Overall, the heatmaps of layer-feature correlations across the 3 modalities not only identify the core layers and key percentile-based features involved in the model’s internal processing, but also provide empirical support for refining noise injection strategies. This contributes to achieving a better balance between privacy protection and model performance.

### Privacy Protection Effect Comparison of Multimodal Methods

To further evaluate the impact of noise addition on model performance, this study uses accuracy as an auxiliary metric, with patients’ survival status as the classification criterion (where deceased patients are labeled as 1 and surviving patients as 0), and implements the classification and prediction of survival status through a neural network with 3 FC layers. Leveraging the pan-cancer dataset, a systematic analysis is conducted on the model’s survival classification performance across 4 data combinations: clinical data, mRNA, miRNA, and CNV. The proposed model is compared with 2 state-of-the-art attention-based models for pan-cancer survival prediction: MultiSurv [17] and Fan et al [10].

**Figure 5:**
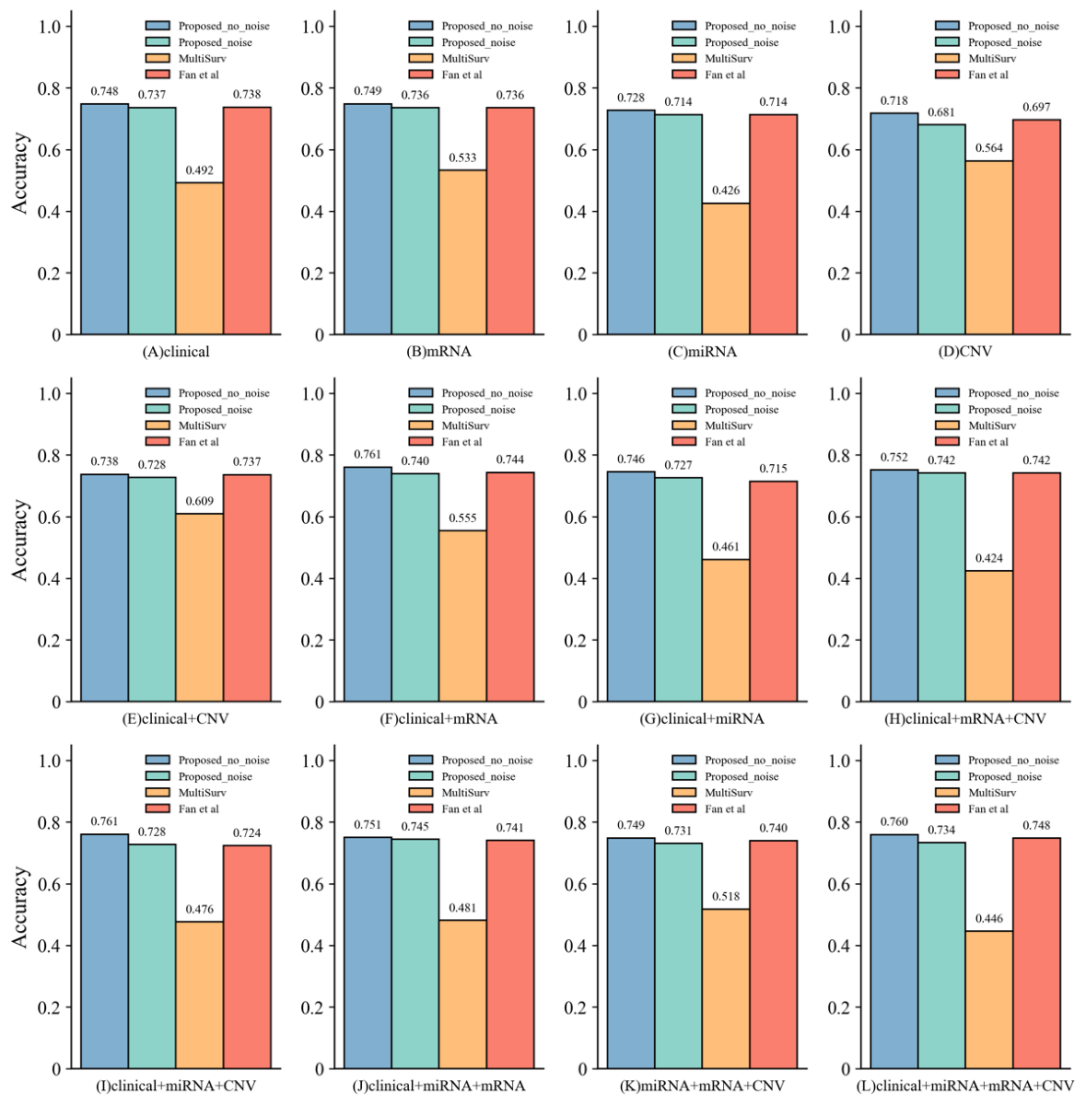
Accuracy comparison results between “Proposed_no_noise” (blue bars), “Proposed method” (green bars), “MultiSurv” (orange bars), and Fan et al [10] (red bars) across (A) clinical, (B) messenger RNA (mRNA), (C) microRNA (miRNA), (D) gene copy number variation (CNV), (E) clinical + CNV, (F) clinical + mRNA, (G) clinical + miRNA, (H) clinical + mRNA + CNV, (I) clinical + miRNA + CNV, (J) clinical + miRNA + mRNA, (K) miRNA + mRNA + CNV, and (L) miRNA + mRNA + CNV modalities.

In unimodal settings, as shown in Figures 5A-5D, the proposed model demonstrates strong robustness. For instance, in the miRNA modality, accuracy slightly decreases from 0.728 before noise injection to 0.714 after; in the mRNA modality, accuracy drops modestly from 0.749 to 0.736.

Experiments involving multimodal combinations were conducted in dual-, tri-, and 4-modality settings. As shown in Figures 5E-5L, the model also maintains stable performance under multimodal conditions. For example, the clinical + miRNA + mRNA combination achieves an accuracy of 0.751 before noise addition and 0.746 afterward; for the clinical + mRNA combination, accuracy declines from 0.761 to 0.740 after noise injection.

### Survival Prediction Using Pan-Cancer Training Dataset

To evaluate the survival prediction advantages of the proposed model across different modalities, this study adopted the C-index as the performance metric and compared it against 6 state-of-the-art models: the CPH [3], RSF [2], MultiSurv, DeepHit [15], CMTA [16], and Fan et al [10]. The experiments are conducted using clinical data, mRNA, miRNA, and CNV data in both unimodal and multimodal settings. All results were obtained via 5-fold cross-validation and are summarized in Table 2.

**Table 2:** Model performance on mixed cancer types using single-modal and multimodal training datasets by the measurements of concordance index (C-index).

Modality	C-index						
	CPH^a^ [3]	RSF^b^ [2]	MultiSurv [17]	DeepHit [15]	CMTA^c^ [16]	Fan et al [10]	Proposed model
Clinical	0.633 (±0.010)	0.745 (±0.008)	0.749 (±0.021)	0.742 (±0.013)	0.758 (±0.009)	0.756 (±0.013)	0.775 (±0.008)^d^
mRNA^e^	0.715 (±0.013)	0.739 (±0.013)	0.747 (±0.012)	0.744 (±0.008)	0.760 (±0.010)	0.764 (±0.013)	0.779 (±0.007)^d^
miRNA^f^	0.715 (±0.010)	0.735 (±0.005)	0.725 (±0.012)	0.701 (±0.008)	0.712 (±0.007)	0.738 (±0.011)	0.760 (±0.011)^d^
CNV^g^	0.589 (±0.008)	0.626 (±0.007)	0.649 (±0.006)	0.579 (±0.006)	0.618 (±0.0011)	0.646 (±0.009)	0.659 (±0.009)^d^
Clinical + CNV	—^h^	—	0.752 (±0.008)	0.747 (±0.006)	0.758 (±0.012)	0.753 (±0.005)	0.763 (±0.006)^d^
Clinical + mRNA	—	—	0.773 (±0.008)	0.764 (±0.011)	0.785 (±0.010)	0.784 (±0.007)	0.792 (±0.006)^d^
Clinical + miRNA	—	—	0.758 (±0.011)	0.760 (±0.008)	0.763 (±0.007)	0.766 (±0.010)	0.783 (±0.007)^d^
Clinical + mRNA + CNV	—	—	0.766 (±0.012)	0.768 (±0.013)	0.774 (±0.013)	0.779 (±0.007)	0.785 (±0.004)^d^
Clinical + miRNA + CNV	—	—	0.760 (±0.009)	0.761 (±0.010)	0.762 (±0.009)	0.764 (±0.007)	0.775 (±0.008)^d^
Clinical + miRNA + mRNA	—	—	0.771 (±0.014)	0.773 (±0.014)	0.779 (±0.013)	0.777 (±0.013)	0.799 (±0.012)^i^
miRNA + mRNA + CNV	—	—	0.747 (±0.013)	0.752 (±0.012)	0.770 (±0.007)	0.767 (±0.008)	0.778 (±0.010)^d^
Clinical + miRNA + mRNA + CNV	—	—	0.768 (±0.013)	0.770 (±0.010)	0.772 (±0.012)	0.779 (±0.010)	0.787 (±0.007)^d^

^a^CPH: Cox proportional hazards model.

^b^RSF: random survival forest.

^c^CMTA: cross-modal translation and alignment.

^d^*P*<.05 vs MultiSurv, DeepHit, CMTA, and Fan et al [10] (paired *t* test with Bonferroni correction, n=1000 bootstrap samples).

^e^mRNA: messenger RNA.

^f^miRNA: microRNA.

^g^CNV: gene copy number variation.

^h^Not available.

^i^*P*<.01 vs CMTA (top competitor; paired *t* test with Bonferroni correction, n=1000 bootstrap samples).

To substantiate claims of superiority despite modest C-index differences, statistical significance testing was performed using bootstrapped resampling and 2-tailed paired *t* tests. For each model and modality combination, 1000 bootstrap samples were generated from the test set. Paired *t* tests were conducted on the bootstrapped C-index values (n=1000) to test the null hypothesis that the mean performance difference is 0, with Bonferroni correction applied for multiple comparisons. Additionally, 95% CIs were derived from the bootstrap distributions. Nonoverlapping CIs are interpreted as indicating statistically significant differences (*P*<.05).

In unimodal prediction scenarios, the proposed model achieves C-index scores of 0.775, 0.779, 0.760, and 0.659 for clinical, mRNA, miRNA, and CNV data, respectively—significantly outperforming the CPH and RSF, which are limited to unimodal survival prediction. Notably, the proposed model yields the highest performance on mRNA data, with a C-index of 0.779, compared to 0.715 for CPH and 0.739 for RSF.

Furthermore, in the multimodal setting, this study compared the performance of MultiSurv, DeepHit, CMTA, Fan et al [10], and the proposed model across various combinations of data modalities. As shown in Table 2, the proposed model consistently achieves C-index values around 0.78 across different combinations. The highest performance is observed in the trimodal configuration (clinical + mRNA + miRNA), which yields a C-index of 0.799—surpassing MultiSurv (0.771), DeepHit (0.773), CMTA (0.779), and Fan et al [10] (0.777). To statistically validate these performance improvements, paired significance testing with multiple-comparison adjustment was conducted. For each model and modality combination, 1000 bootstrap samples were generated from the test set. Paired *t* tests were performed on the bootstrapped C-index values (n=1000) with Bonferroni correction applied for multiple comparisons. The proposed model demonstrated statistically significant improvements over all competitors in the optimal trimodal configuration (clinical + mRNA + miRNA) after multiplicity adjustment: vs CMTA (*P*=.008), Fan et al [10] (*P*=.006), MultiSurv (*P*=.003), and DeepHit (*P*=.004). These adjusted *P* values (<.05) confirm that the observed C-index improvements (0.020, 0.022, 0.028, and 0.026 relative to CMTA, Fan et al [10], MultiSurv, and DeepHit, respectively) are statistically robust and not attributable to random variation.

This superior performance is attributed to the model’s dual-layer feature extraction architecture, which, leveraging multiview parallel processing, enables finer-grained automatic weight adjustment. Notably, our proposed model further integrates an adaptive DP protection mechanism to safeguard sensitive medical data, while still achieving a higher C-index compared to existing state-of-the-art multimodal models that lack privacy-preserving designs. These findings reinforce the advantage of multimodal inputs over unimodal inputs, as multimodal data provide more comprehensive and informative cues for survival prediction.

### Comparison With Standard Privacy Protection Methods

To comprehensively validate the superiority of the proposed adaptive DP framework in balancing privacy protection and predictive utility, this study adopted 2 core metrics—C-index for survival outcome ranking accuracy and classification accuracy for binary survival status prediction (deceased=1 and surviving=0)—and systematically compared it against 3 standard DP-aware baseline models: DP-Stochastic Gradient Descent (SGD) [36], DP-Adam [37], and Private Aggregation of Teacher Ensembles (PATE) [38]. The experiments were conducted under the optimal multimodal setting (clinical + mRNA + miRNA), with a fixed global privacy budget of ϵ = 0.8 (δ = 1e-5 for DP-SGD and DP-Adam, as per their standard implementations) to ensure a fair comparison of use under identical formal privacy guarantees. Consistent data preprocessing (zero-vector imputation for missing modalities, minimum-maximum normalization) and model training configurations (Adam optimizer, learning rate=0.001, batch size=256, and 100 epochs) were used across all methods to eliminate confounding variables. All results were derived from 5-fold cross-validation on the pan-cancer test set and supplemented with 1000 bootstrap resamples for statistical significance testing (paired *t* test with Bonferroni correction, *P*<.05). Detailed performance metrics and comparative analysis are summarized in Table 3.

Table 3 clearly shows that the proposed adaptive DP framework outperforms all standard DP baselines in maintaining predictive utility under the same formal privacy guarantee (ϵ=0.8). Regarding the C-index—the gold standard for survival prediction that assesses the ordinal consistency between predicted risk scores and actual survival times—the proposed model achieves 0.799 (±0.012), which is 4.3, 3.7, and 6.5 percentage points higher than DP-SGD (0.756 ± 0.015), DP-Adam (0.762 ± 0.014), and PATE (0.734 ± 0.016), respectively. Its utility loss compared to the non-DP counterpart (0.821 ± 0.008) is only 0.022, which is less than half of DP-SGD’s loss (0.065) and about 1/3 of PATE’s loss (0.087). This confirms that the layer-wise relevance propagation–guided targeted noise injection strategy more efficiently uses the privacy budget, preserving high-relevance prognostic features while satisfying ϵ-DP.

In terms of binary survival status classification accuracy, the proposed model maintains a high level of 0.745 (±0.013), outperforming the aforementioned baselines by 0.8, 1.0, and 1.4 percentage points, respectively. Its accuracy drop (0.006) is also significantly smaller than that of the baselines (0.014-0.020), further validating the efficiency of adaptive noise allocation in minimizing utility degradation for a given ϵ. Statistical analysis (paired *t* test with Bonferroni correction, n=1000 bootstrap samples) reveals that the proposed model’s C-index and accuracy are significantly different from all baselines (*P*<.05), with nonoverlapping 95% CIs, confirming the robustness of the observed improvements.

**Table 3:** Use comparison of proposed model vs standard differential privacy (DP) baselines (multimodal: clinical + messenger RNA + microRNA; ϵ=0.8).

Modality	C-index^a^	Accuracy
Proposed_no_noise	0.821 (±0.008)	0.751 (±0.008)
Proposed model	0.799 (±0.012)	0.745 (±0.013)
DP-SGD^b^	0.756 (±0.015)	0.737 (±0.010)
DP-Adam	0.762 (±0.014)	0.735 (±0.008)
PATE^c^	0.734 (±0.016)	0.731 (±0.009)

^a^C-index: concordance index.

^b^SGD: Stochastic Gradient Descent

^c^PATE: Private Aggregation of Teacher Ensembles.

### Analysis of Privacy Protection Effectiveness

The proposed adaptive DP mechanism is designed to satisfy ϵ-DP (ϵ=0.8) as formally proven in equation 20. Compared with traditional uniform noise injection methods (DP-SGD and DP-Adam) under the same global privacy budget, our method significantly improves the accuracy of survival prediction (with the C-index increased by 3.7-6.5 percentage points; Table 3). This result indicates that, for the same formal privacy guarantee (ϵ), our adaptive allocation strategy achieves higher predictive utility. The adaptive mechanism identifies features with high relevance to prediction and allocates a larger portion of the privacy budget to them (injecting less noise), thereby preserving critical prognostic information. Conversely, features with low predictive contribution receive a smaller budget (more noise), which theoretically enhances privacy protection for those elements without harming overall model utility. Therefore, the proposed method provides a stronger formal privacy guarantee for low-relevance features and achieves superior utility for high-relevance features under the same ϵ constraint, representing a more efficient use of the privacy budget.

### Comparison of Survival Prediction Performance for Individual Cancer Types

**Figure 6:**
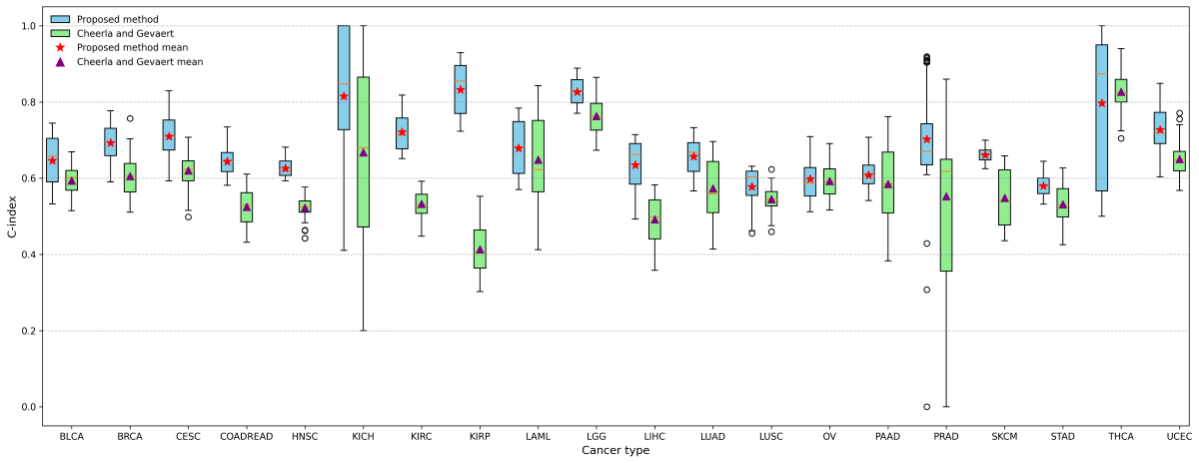
Concordance index (C-index) of the proposed model (blue bars) and the previous work (Cheerla and Gevaert [9]; green bars) on the 20 cancer types using the modality combination of clinical, microRNA, and messenger RNA. BLCA: bladder urothelial carcinoma; BRCA: breast invasive carcinoma; CESC: cervical squamous cell carcinoma; COADREAD: colon adenocarcinoma/rectum adenocarcinoma; HNSC: head and neck squamous cell carcinoma; KICH: kidney chromophobe; KIRC: kidney renal clear cell carcinoma; KIRP: kidney renal papillary cell carcinoma; LAML: acute myeloid leukemia; LGG: low-grade glioma; LIHC: liver hepatocellular carcinoma; LUAD: lung adenocarcinoma; LUSC: lung squamous cell carcinoma; OV: ovarian serous cystadenocarcinoma; PAAD: pancreatic adenocarcinoma; PRAD: prostate adenocarcinoma; SKCM: skin cutaneous melanoma; STAD: stomach adenocarcinoma; THCA: thyroid carcinoma; UCEC: uterine corpus endometrial carcinoma.

To investigate the effectiveness of the proposed model in predicting survival outcomes for individual cancer types, this study conducted a comparison between our proposed model and the state-of-the-art deep learning–based model (Cheerla and Gevaert [9]) for single-cancer prediction using pan-cancer datasets. For consistency, this study adopted a multimodal input comprising clinical, miRNA, and mRNA data. The results are presented in Figure 6.

Compared with Cheerla and Gevaert [9], our proposed method achieved a higher C-index in the majority of cancer types—18 out of 20. Notably, in 7 of these 18 cancer types, the performance improvement was substantial, with C-index differences exceeding 0.1.

Furthermore, as illustrated in Figure 6, our model exhibits higher median C-index values for several cancer types and demonstrates more concentrated distributions in the upper range. This indicates both superior and more stable predictive performance. In contrast, Cheerla and Gevaert [9] show relatively lower C-index scores in some cancer types, with several boxplots revealing greater data dispersion and reduced stability.

### Comparison Between Using Single-Cancer and Pan-Cancer Training Datasets

To further investigate the predictive performance of the proposed model across different types of datasets, this study conducted experiments using both pan-cancer and single-cancer datasets. The first objective was to examine whether training on pan-cancer data improves survival prediction accuracy for individual cancer types. In this study, we selected the same 20 cancer types as those used by Cheerla and Gevaert [9], where patients have significantly different survival patterns.

**Figure 7:**
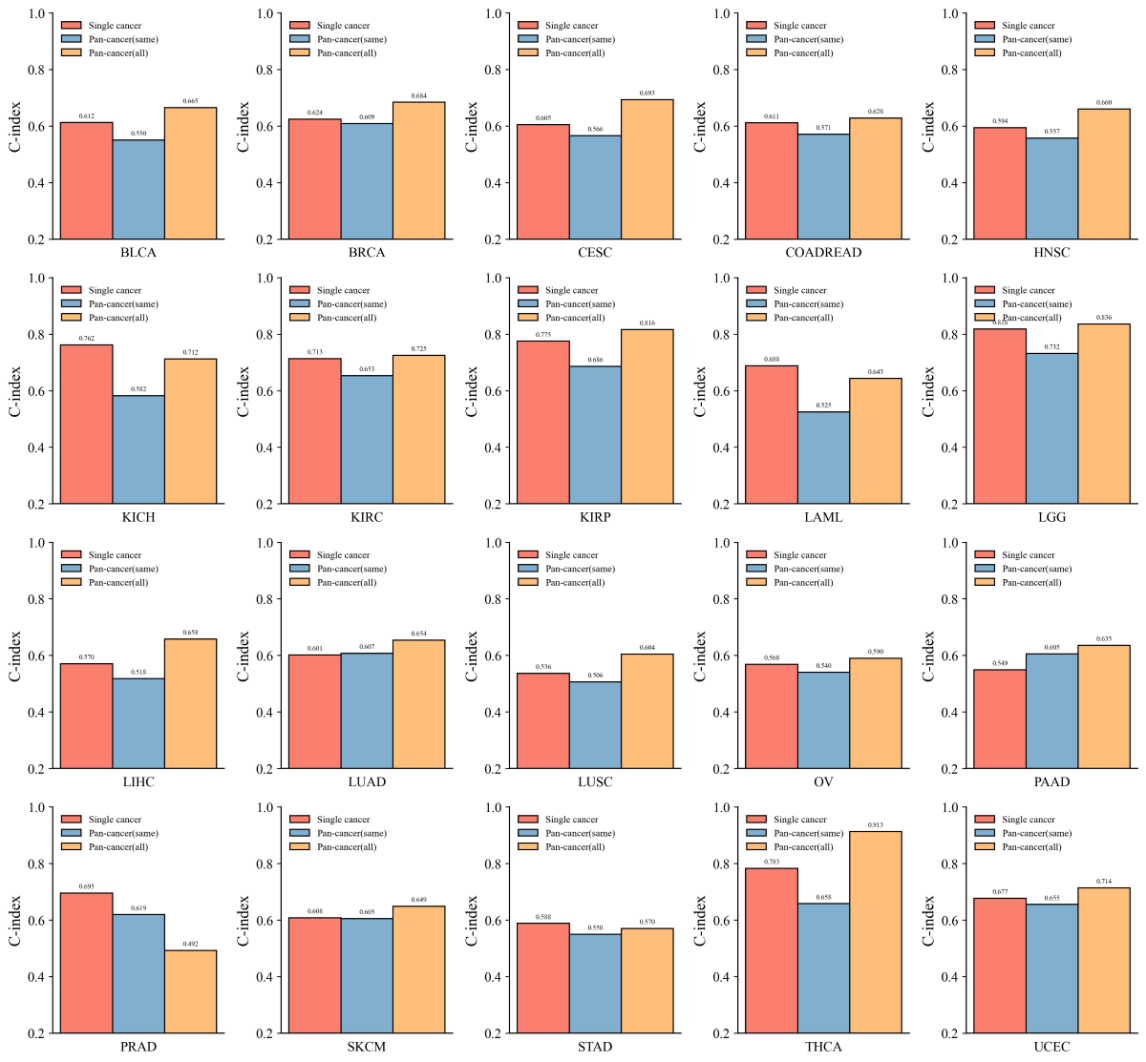
Concordance index (C-index) scores of the proposed model trained on single-cancer and pan-cancer datasets on 20 cancer types using clinical, messenger RNA, and microRNA modalities. Red bars represent models trained on single-cancer datasets, where only patients with the same cancer type were used. Blue bars indicate models trained on pan-cancer datasets with sample sizes matched to each single-cancer dataset to control for sample size bias. Orange bars correspond to models trained on the full pan-cancer dataset. BLCA: bladder urothelial carcinoma; BRCA: breast invasive carcinoma; CESC: cervical squamous cell carcinoma; COADREAD: colon adenocarcinoma/rectum adenocarcinoma; HNSC: head and neck squamous cell carcinoma; KICH: kidney chromophobe; KIRC: kidney renal clear cell carcinoma; KIRP: kidney renal papillary cell carcinoma; LAML: acute myeloid leukemia; LGG: low-grade glioma; LIHC: liver hepatocellular carcinoma; LUAD: lung adenocarcinoma; LUSC: lung squamous cell carcinoma; OV: ovarian serous cystadenocarcinoma; PAAD: pancreatic adenocarcinoma; PRAD: prostate adenocarcinoma; SKCM: skin cutaneous melanoma; STAD: stomach adenocarcinoma; THCA: thyroid carcinoma; UCEC: uterine corpus endometrial carcinoma.

This study first compared the performance of models trained solely on single-cancer data with those trained on all pan-cancer samples, using consistent test sets for each cancer type. For single-cancer experiments, this study selected patients with the same cancer type in pan-cancer training-validation-test sets to form the single-cancer training-validation-test sets. The model was trained using integrated clinical, mRNA, and miRNA modalities, and the results are shown in Figure 7.

As shown by the bar plots labeled “Single cancer” and “Pan-cancer (all)” in Figure 7, the model trained on pan-cancer data generally outperformed the single-cancer–trained model on the same test sets, with the exception of 4 cancer types: kidney chromophobe, acute myeloid leukemia, prostate adenocarcinoma, and stomach adenocarcinoma. For example, in the case of bladder urothelial carcinoma the pan-cancer–trained model achieved a C-index of 0.665, significantly higher than the 0.612 achieved by the model trained only on bladder urothelial carcinoma data.

To control for potential biases due to varying training sample sizes, this study further evaluated model performance when trained on an equal number of samples from the pan-cancer and single-cancer datasets. Specifically, we selected subsets of pan-cancer data matching the sample size of each individual cancer dataset. As illustrated by the bar plots labeled “Single cancer” and “Pan-cancer (same)” in Figure 7, the single-cancer–trained models generally outperformed the pan-cancer–trained counterparts under equal training size conditions.

**Figure 34:**
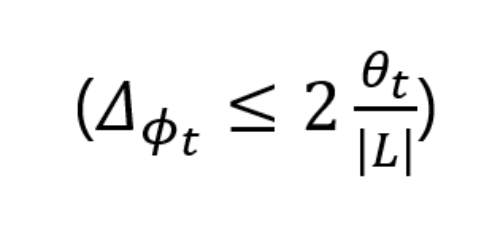
Inline graphic 27.

### External Validation on Independent Cohorts

To address the critical question of generalizability beyond the TCGA dataset, we conducted an external validation on 2 independent cancer cohorts from the International Cancer Genome Consortium (ICGC) database [39]. Specifically, we selected the hepatocellular carcinoma (HCC; ICGC-HCC) and clear cell renal cell carcinoma (ccRCC; ICGC-ccRCC) projects, which provide clinical survival information and mRNA expression data compatible with our model’s input requirements. To maintain consistency with our multimodal input configuration (clinical + mRNA + miRNA), the same zero-vector imputation strategy described in the Methods section (“Data Preprocessing”) was applied to handle the missing miRNA modality and any mRNA features absent in the external data. The ICGC-HCC cohort comprised 242 patients with overall survival data, and the ICGC-ccRCC cohort comprised 530 patients. For each cohort, we preprocessed the mRNA expression data by aligning gene features to the 1579 mRNA features used in our TCGA training set. All continuous features were normalized using the minimum and maximum values derived from the TCGA training set to ensure consistent scaling.

This study applied our proposed model (trained on the pan-cancer TCGA dataset with clinical + mRNA + miRNA modalities) directly to the preprocessed ICGC cohorts without any retraining or fine-tuning. The model’s survival prediction performance was evaluated using the C-index.

Our proposed model, trained on the pan-cancer TCGA dataset, achieved a C-index of 0.721 on the independent ICGC-HCC cohort and 0.708 on the ICGC-ccRCC cohort. This demonstrates that the model maintains its generalizable prognostic discriminatory power on unseen, external patient populations.

## Discussion

### Principal Results

In this study, we evaluated the model’s classification performance in survival prediction across 4 data combinations—clinical data, mRNA, miRNA, and CNV—using the pan-cancer dataset. Compared with MultiSurv [17], the proposed model exhibits an approximately 0.3 improvement in prediction accuracy across all modality combinations after noise injection. When compared with Fan et al [10], the proposed model achieves higher accuracy in all settings before noise addition, and maintains comparable accuracy after noise injection, despite the latter model not incorporating any privacy-preserving mechanisms.

The improvement in survival prediction across different modalities stems from the model’s ability to capture key information from different subspaces in parallel, thereby overcoming the representational limitations of single-attention mechanisms. By effectively integrating core features from each modality, the model enhances the flexibility and expressiveness of the fusion process. Notably, the inclusion of the CNV modality generally leads to a decline in predictive performance, suggesting that CNV data contain relatively fewer informative features relevant to patients’ survival. This further underscores the value of multimodal data fusion in enhancing the generalizability of survival prediction models.

Comparisons in single-cancer survival prediction further demonstrate that the model achieves higher and more stable C-index values in 18 out of 20 cancer types compared with existing deep learning methods, highlighting its robustness in individualized survival prediction. Our study not only confirms the effectiveness and superiority of the proposed model in individualized cancer survival prediction but also demonstrates its ability to preserve patients’ privacy. Collectively, these results provide a more reliable and privacy-conscious solution for precision oncology applications.

In addition, experiments conducted on both pan-cancer and single-cancer datasets show that the proposed model maintains strong predictive capability across diverse data settings, demonstrating robust generalization ability.

### Challenges

While the model demonstrates strong privacy-preserving capabilities through DP, the integration of DP mechanisms into clinical artificial intelligence models introduces certain challenges. First, DP may impact the interpretability of the model, as the noise added to the gradients or model parameters can obscure the relationships between input features and predicted outcomes. This could reduce the transparency of the model, making it more difficult for clinicians to trust and understand the model’s decisions, which is critical in medical applications. In addition, DP mechanisms may complicate auditability, as it becomes harder to trace the influence of individual data points on the model’s predictions due to the noise injected for privacy protection.

Second, the integration of privacy mechanisms such as DP into clinical workflows or federated research environments can be practically challenging. In clinical settings, where data are highly sensitive, ensuring that privacy is preserved while maintaining the usability and accuracy of predictive models requires careful consideration of computational resources and model deployment strategies. In federated research environments, where data are distributed across multiple institutions, ensuring that DP mechanisms are applied consistently across all sites without compromising the model’s predictive performance can be technically difficult. Moreover, adapting these privacy-preserving mechanisms to existing clinical systems and ensuring compliance with data protection regulations (eg, General Data Protection Regulation requires close collaboration with regulatory bodies and careful planning of data-sharing protocols.

These considerations highlight the need for further research into improving the balance between privacy protection, model interpretability, and practical integration in clinical and research settings.

### Limitations

The proposed model has certain limitations. First, while we have demonstrated the model’s effectiveness on the TCGA pan-cancer dataset and provided initial evidence of generalizability through external validation on ICGC cohorts, the performance on independent datasets shows a nonnegligible decline. This underscores the inherent challenge of translating models trained on 1 cohort (even a large and diverse one like TCGA) to others due to population, treatment, and technical variability. More extensive validation across multiple, prospectively collected cohorts is needed to fully establish clinical readiness. For example, training efficiency requires improvement. Analysis suggests that one possible reason is the introduction of the bilayer feature extraction module based on MHA and the adaptive DP protection mechanism, which increases algorithmic computational complexity. Additionally, the model exhibits increased inference time compared with simpler architectures such as CPH or RSF, due to the MHA mechanism and the privacy-preserving noise injection process. This may limit its deployment in real-time clinical settings where rapid predictions are required. Meanwhile, comparative experiments with other advanced models on larger-scale multimodal datasets are necessary. Indeed, in the era of big data and multimodal data fusion, new challenges lie ahead.

### Future Work

Future work will expand in several directions. On the one hand, integrating pathological images and proteomics data will enhance multimodal feature fusion, extracting and fusing more information to further improve prediction performance. On the other hand, the model will be applied to additional tasks such as cancer subtype classification and biomarker discovery. Finally, research on other noise mechanisms based on DP is needed to protect sensitive information from multiple perspectives and ensure the security of model training and sharing. Moreover, efforts will be made to optimize the computational efficiency and reduce inference time through techniques such as model pruning, quantization, or lightweight attention mechanisms, thereby enhancing the model’s practical applicability in clinical environments. To address potential limitations of zero-vector imputation, future work will also explore advanced missing-modality handling strategies, including masking strategies and missing-modality–aware attention mechanisms.

### Conclusions

To address the key challenges of inefficient multimodal integration, insufficient privacy protection, and limited generalizability in pan-cancer survival prediction, this study proposes a bilayer feature fusion model integrating the MHA mechanism and adaptive DP. Innovatively, the framework uses modality-specific FC networks in the first layer to extract features from clinical, mRNA, miRNA, and CNV data. The second layer uses MHA to model cross-modal interactions through parallel attention heads, dynamically allocating weights to focus on survival-related features and overcoming the limitations of static fusion. Additionally, layer-wise relevance analysis is used during first-layer feature extraction to quantify the correlation between features and outcomes, enabling adaptive DP—injecting less Laplacian noise into the gradients of high-correlation features and more noise into low-correlation features to balance privacy and utility. Experimental results on pan-cancer datasets from TCGA demonstrate the feasibility and superiority of our proposed method. Furthermore, external validation on independent ICGC cohorts provided preliminary evidence of the model’s generalizability, showing retained predictive power superior to clinical-only models despite expected performance attenuation due to cohort heterogeneity.

## Abbreviations

ccRCC: clear cell renal cell carcinoma
C-index: concordance index
CMTA: cross-modal translation and alignment
CNV: gene copy number variation
CPH: Cox proportional hazards model
DP: differential privacy
FC: fully connected
HCC: hepatocellular carcinoma
ICGC: International Cancer Genome Consortium
MHA: multihead attention
miRNA: microRNA
mRNA: messenger RNA
PATE: Private Aggregation of Teacher Ensembles
RSF: random survival forest
SGD: Stochastic Gradient Descent
TCGA: The Cancer Genome Atlas

## Data Availability

The data that support the findings of this study are openly available in the Xena database [40] and the TCGA database [41].

## References

[1] Vale-Silva LA, Rohr K (2021). Long-term cancer survival prediction using multimodal deep learning. Sci Rep.

[2] Ishwaran H, Kogalur UB, Blackstone EH, Lauer MS (2008). Random survival forests. Ann. Appl. Stat.

[3] Cox DR (2018). Regression models and life-tables. Journal of the Royal Statistical Society: Series B (Methodological).

[4] Katzman JL, Shaham U, Cloninger A, Bates J, Jiang T, Kluger Y (2018). DeepSurv: Personalized treatment recommender system using a Cox proportional hazards deep neural network. BMC Med Res Methodol.

[5] Kim DW, Lee S, Kwon S, Nam W, Cha I, Kim HJ (2019). Deep learning-based survival prediction of oral cancer patients. Sci Rep.

[6] Wulczyn E, Steiner DF, Moran M, Plass M, Reihs R, Tan F, Flament-Auvigne I, Brown T, Regitnig P, Chen PC, Hegde N, Sadhwani A, MacDonald R, Ayalew B, Corrado GS, Peng LH, Tse D, Müller H, Xu Z, Liu Y, Stumpe MC, Zatloukal K, Mermel CH (2021). Interpretable survival prediction for colorectal cancer using deep learning. NPJ Digit Med.

[7] Wulczyn E, Steiner DF, Xu Z, Sadhwani A, Wang H, Flament-Auvigne I, Mermel CH, Chen PC, Liu Y, Stumpe MC (2020). Deep learning-based survival prediction for multiple cancer types using histopathology images. PLoS One.

[8] Yeoh PSQ, Lai KW, Goh SL, Hasikin K, Wu X, Li P (2023). Transfer learning-assisted 3D deep learning models for knee osteoarthritis detection: Data from the osteoarthritis initiative. Front Bioeng Biotechnol.

[9] Cheerla A, Gevaert O (2019). Deep learning with multimodal representation for pancancer prognosis prediction. Bioinformatics.

[10] Fan Z, Jiang Z, Liang H, Han C (2023). Pancancer survival prediction using a deep learning architecture with multimodal representation and integration. Bioinform Adv.

[11] Guo W, Liang W, Deng Q, Zou X (2021). A multimodal affinity fusion network for predicting the survival of breast cancer patients. Front Genet.

[12] Tan K, Huang W, Liu X, Hu J, Dong S (2022). A multi-modal fusion framework based on multi-task correlation learning for cancer prognosis prediction. Artif Intell Med.

[13] Na H, Wang L, Zhuang X (2023). Attention-based multimodal bilinear feature fusion for lung cancer survival analysis.

[14] Li R, Wu X, Li A, Wang M (2022). HFBSurv: Hierarchical multimodal fusion with factorized bilinear models for cancer survival prediction. Bioinformatics.

[15] Lee C, Yoon J, Schaar MVD (2020). Dynamic-deepHit: A deep learning approach for dynamic survival analysis with competing risks based on longitudinal data. IEEE Trans Biomed Eng.

[16] Kim T, Cho H, Yoon KJ (2024). Cmta: Cross-modal temporal alignment for event-guided video deblurring.

[17] Silva LAV, Rohr K (2020). Pan-cancer prognosis prediction using multimodal deep learning.

[18] Zhang Z, Yin W, Wang S, Zheng X, Dong S (2024). MBFusion: Multi-modal balanced fusion and multi-task learning for cancer diagnosis and prognosis. Comput Biol Med.

[19] Wang C, Guo J, Zhao N, Liu Y, Liu X, Liu G, Guo M (2020). A cancer survival prediction method based on graph convolutional network. IEEE Trans Nanobioscience.

[20] Flack D, Tripathi A, Waqas A, Rasool G, Dera D (2025). Robust multimodal fusion for survival prediction in cancer patients. Cancer Inform.

[21] Venkatesaramani R, Malin BA, Vorobeychik Y (2021). Re-identification of individuals in genomic datasets using public face images. Sci Adv.

[22] Kaissis GA, Makowski MR, Rückert D, Braren RF (2020). Secure, privacy-preserving and federated machine learning in medical imaging. Nat Mach Intell.

[23] Chen H, Wang N, Zhou Y, Mei K, Tang M, Cai G (2023). Breast cancer prediction based on differential privacy and logistic regression optimization model. Applied Sciences.

[24] Chai H, Huang Y, Xu L, Song X, He M, Wang Q (2024). A decentralized federated learning-based cancer survival prediction method with privacy protection. Heliyon.

[25] Wu X, Wei Y, Mao Y, Wang L (2018). A differential privacy DNA motif finding method based on closed frequent patterns. Cluster Comput.

[26] Wu X, Zhang Y, Shi M, Li P, Li R, Xiong NN (2022). An adaptive federated learning scheme with differential privacy preserving. Future Generation Computer Systems.

[27] Wang H, Zhang X, Xia Y, Wu X (2023). An intelligent blockchain-based access control framework with federated learning for genome-wide association studies. Computer Standards & Interfaces.

[28] Wang H, Zhang X, Xia Y, Wu X (2023). An intelligent blockchain-based access control framework with federated learning for genome-wide association studies. Computer Standards & Interfaces.

[29] Wang H, Wu X (2023). IPP: An intelligent privacy-preserving scheme for detecting interactions in genome association studies. IEEE/ACM Trans Comput Biol Bioinform.

[30] Al Fatih Abil Fida M, Ahmad T, Ntahobari M (2021). Variance threshold as early screening to boruta feature selection for intrusion detection system.

[31] Gajera V, Gupta R, Jana PK (2016). An effective multi-objective task scheduling algorithm using min-max normalization in cloud computing.

[32] Hutter C, Zenklusen JC (2018). The cancer genome atlas: Creating lasting value beyond its data. Cell.

[33] Malta TM, Sokolov A, Gentles AJ, Burzykowski T, Poisson L, Weinstein JN, Kamińska B, Huelsken J, Omberg L, Gevaert O, Colaprico A, Czerwińska P, Mazurek S, Mishra L, Heyn H, Krasnitz A, Godwin AK, Lazar AJ, Stuart JM, Hoadley KA, Laird PW, Noushmehr H, Wiznerowicz M, Cancer Genome Atlas Research Network (2018). Machine learning identifies stemness features associated with oncogenic dedifferentiation. Cell.

[34] Ioffe S, Szegedy C (2015). Batch normalization: Accelerating deep network training by reducing internal covariate shift. arXiv:1502.03167.

[35] Harrell FE, Califf RM, Pryor DB, Lee KL, Rosati RA (1982). Evaluating the yield of medical tests. JAMA.

[36] Abadi M, Chu A, Goodfellow I, McMahan HB, Mironov I, Talwar K (2016). Deep learning with differential privacy.

[37] Tang Q, Lécuyer M (2023). DP-Adam: Correcting DP bias in adam's second moment estimation. arXiv:2304.11208.

[38] Papernot N, Abadi M, Erlingsson U, Goodfellow I, Talwar K (2016). Semi-supervised knowledge transfer for deep learning from private training data. arXiv:1610.05755.

[39] ICGC ARGO.

[40] UCSC Xena.

[41] GDC Data Portal.

